# *DNM1L*-Related Mitochondrial Fission Defects Presenting as Encephalopathy: A Case Report and Literature Review

**DOI:** 10.3389/fped.2021.626657

**Published:** 2021-07-08

**Authors:** Xingmiao Liu, Zhongbin Zhang, Dong Li, Meifang Lei, Qing Li, Xiaojun Liu, Peiyuan Zhang

**Affiliations:** Department of Pediatric Neurology, Tianjin Children's Hospital/Tianjin University Children's Hospital, Tianjin, China

**Keywords:** *DNM1L*, DRP1, mitochondrial disease, psychomotor retardation, dyskinesia, hypertonia, follow-up study

## Abstract

**Background:** Mitochondrial dynamics, including mitochondrial fission and fusion, transport and distribution, biogenesis and degradation, are critical to neuronal function. The dynamin-1 like *(DNM1L)* gene encodes dynamin-related protein 1 (DRP1/DLP1), which is an evolutionarily conserved member of the dynamin family and is responsible for mitochondrial division. *DNM1L* variants can lead to mitochondrial fission dysfunction and neurological disorders.

**Methods:** We report a case of *DNM1L*-related mitochondrial disease admitted to Tianjin Children's Hospital. We searched for similar reported cases in the PubMed database using the terms “*DNM1L*” and “mitochondrial,” reviewed recent literature to summarize the clinical and genetic characteristics, and analyzed genotype-phenotype correlations.

**Results:** The patient presented with psychomotor retardation, motor disturbance (muscle weakness with paroxysmal hypermyotonia), and a *de novo* variant (c.116G>A, g.22229G>A, p.S39N) in the GTPase domain of *DNM1L* (reference sequence NM_012062), which has not previously been reported in the literature. This case was combined with an additional 35 cases identified in 20 relevant references in order to analyze a total of 36 patients. The male-to-female ratio was 1:1.06, and the median age of onset was 6 months (range, neonatal period to 9 years). The cardinal symptoms included psychomotor retardation in 77.8% (28/36), limb paralysis in 66.7% (18/27), dystonia in 82.8% (24/29), and epilepsy in 59.4% (19/32). The clinical manifestations of variants in the GTPase domain of DRP1 were milder than those identified in the middle domain.

**Conclusion:** This case report describes a new variant of the *DNM1L* gene, and summarizes previously reported cases. Furthermore, the clinical phenotype and the genotype of *DNM1L* gene-associated mitochondrial disease was analyzed to improve the understanding of this disease.

## Introduction

Mitochondrial diseases are a group of multi-system disorders with heterogeneous clinical manifestations and genetic characteristics. They are defined by mitochondrial structural abnormalities and dysfunction caused by variants in mitochondrial DNA or nuclear DNA (1). In addition to structural defects in the mitochondrial respiratory chain complex and mitochondria, mutations affecting a group of genes enconding for mitochondrial dynamics-related proteins, which act on mitochondrial fusion, fission, transport, distribution, biogenesis, degradation, and for other related proteins, can also lead to mitochondrial dysfunction (2). Mitochondrial fission and fusion play vital roles in maintaining functional mitochondria when cells encounter environmental and metabolic stresses. Mitochondrial fission can create new mitochondria, but it also contributes to quality control by enabling the removal of damaged mitochondria and facilitating apoptosis to maintain metabolism during cellular stress. Disruptions in these processes affect normal physiological processes and have been implicated in mitochondrial diseases (3).

The most important mediator of mitochondrial and peroxisome division is DRP1, which is encoded by the *DNM1L* gene (4, 5). DRP1 is recruited from the cytosol to the outer mitochondrial membrane, where it oligomerizes, hydrolyzes guanosine triphosphate (GTP), and forms spirals around the mitochondria to constrict both outer and inner membranes to complete mitochondrial fission (3, 6).

The neurological disorder associated with *DNM1L* variants is referred to as encephalopathy due to defective mitochondrial and peroxisomal fission (EMPF1), which has been described as a fatal encephalopathy (7). EMPF1 is characterized by the early onset of a range of symptoms, including psychomotor delay, limb paralysis, dystonia (hypertonia or hypotonia), epilepsy (most refractory seizures, status epilepticus, consistent with epileptic encephalopathy), ataxia, nystagmus, optic atrophy, nystagmus, dysarthria, microcephalus, pain insensitivity, sensory and motor axonal neuropathy, respiratory distress, and even death in childhood.

There have been relatively few reports on *DNM1L*-related mitochondrial diseases; moreover, most of the reported cases are in medical records, which lack details of the disease characteristics. Here, we report a case of *DNM1L*-related mitochondrial disease admitted to Tianjin Children's Hospital and review recent literature to summarize the clinical and genetic characteristics, as well as the phenotype-genotype correlation to deepen our understanding of this genetic disease.

## Clinical Report

### Case Presentation

#### Clinical Manifestation

**Figure 1:**
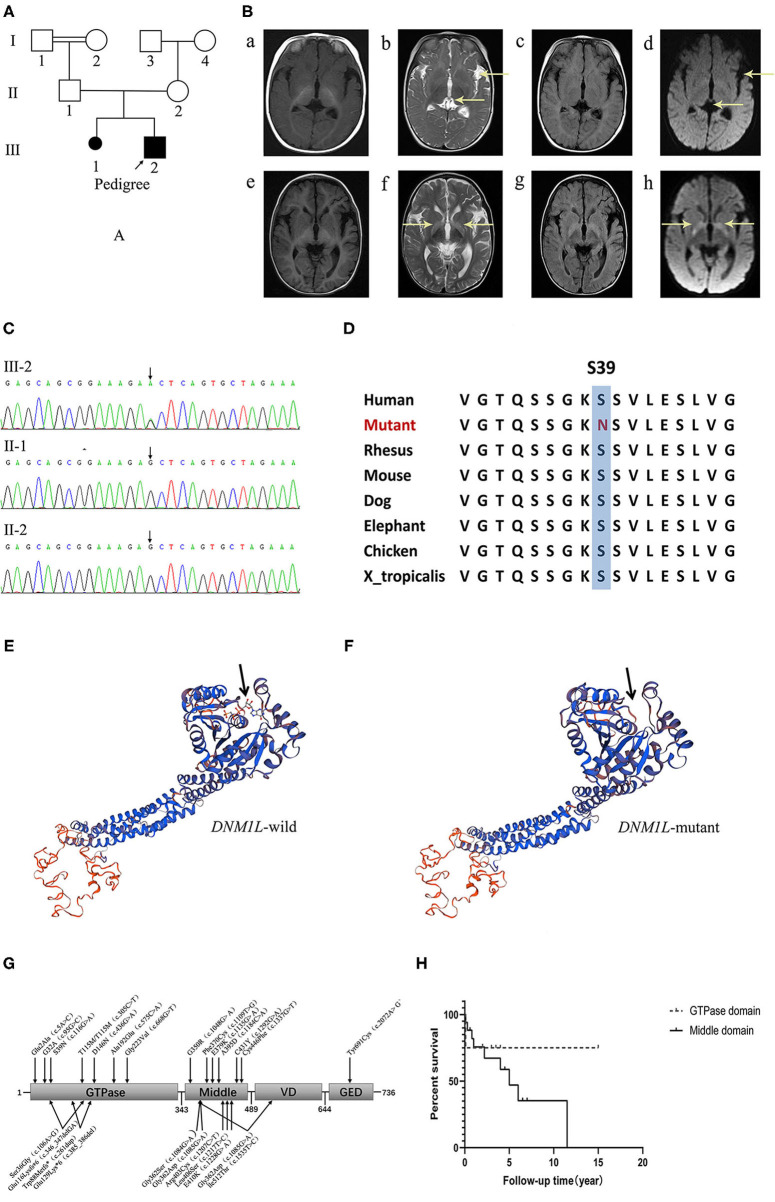
**(A)** The pedigree of the family. **(B)** Brain MR images at 5-, and 18-months of age. (a–d) show that the ventricles of the brain and the left lateral fissure were widened on T1WI, T2WI, FLAIR and DWI sequences at 5-months of age. (e–h) disclose symmetrical hyperintensity in the bilateral basal ganglia on T2WI, FLAIR, and DWI sequences at 18-months of age. **(C)** Electropherograms of the regions containing the *DNM1L* variant identified in genomic DNA from the patient. **(D)** The conservation of the identified amino acid across different species. **(E,F)** The protein 3D modeling for the *DNM1L*-wild and identified missense variant. **(G)** The schematic chart and the locations of identified variants in DRP1. **(H)** The survival curve of patients of the GTPase domain group and the middle domain group of DRP1.

The patient, a 3-year-old boy, was born at term through an uneventful delivery with normal birth parameters of consanguineous Chinese parents. He was the first child in a second pregnancy, after the first resulted in an induced abortion due to fetal death. The grandparents of the patients were cousins. The pedigree of the family was as follows (Figure 1A). The age at the first visit was 5 months. The first symptoms observed were motor and cognitive retardation. He could not attain head control, turn over, or actively grasp objects. He had normal eyesight and hearing. The second symptom, paroxysmal hypertonia, began as early as 1 month after birth. Initially, the symptoms were clenched fists with both hands and straightened limbs, which gradually progressed to flexion of both upper limbs, fisted hands, and straightened lower limbs. During the course, consciousness was clear and the symptoms lasted for a few seconds to a few minutes before spontaneous relief. Physical examination revealed microcephaly, white skin, yellow hair, slightly long face, high palatine arch, right hand penetration, small penis, and small hands and feet. His muscle force was grade three, and he had paroxysmal hypertonia, negative limb deep reflection, and bilateral symmetric flexor plantar reflex.

#### Auxiliary Examination

Magnetic resonance imaging (MRI) showed ventricular broadening and left lateral fissure widening in 2017. 1 year later, MRI revealed symmetric hyperintensity in the bilateral basal ganglia on T2-weighted imaging, T2-FLAIR, and DWI sequences (Figure 1B). Video electroencephalogram showed a normal background and recorded paroxysmal limb rigidity with no epileptiform discharges. Liver and renal function, myocardial enzyme, and ceruloplasmin levels were normal, but metabolism screening showed mild ketonuria and hyperlactacidemia (2.55–3.36). G-banding karyotype analysis: 46, XY. Gesell Developmental Scale: Adaptability: DQ 26.7, DA 2.5, severe delay; Gross motor: DQ 23.7, DA 2.2, extremely severe delay; Fine motor: DQ 24.7, DA 2.3, extremely severe delay; Communication: DQ 49.5, DA 4.6, moderately delayed; personal-social skills: DQ 37.6, DA 3.5, severe delay.

#### Genetic Analysis

DNA was extracted from peripheral blood using the QIAamp DNA Blood Mini Kit (QIAGEN). A total of 3 μg of patient DNA was sheared to produce 200–300 base pair (bp) fragments. Whole-exome enrichment was performed using an Agilent SureSelect Target Enrichment system (Agilent Technologies). Coding exons and flanking intronic regions were enriched with the Agilent SureSelect Human All Exon V6 reagent (Agilent Technologies), according to the manufacturer's protocol. Captured libraries were loaded onto a HiSeq 2500 platform (Illumina). Base calling and assessments of sequence read quality were performed using Illumina Sequence Control Software (SCS; Illumina), which provided real-time analyses. Reads with average quality scores <25 were removed, and bases with quality scores <20 were trimmed. The mean read depth of each sample was 100 ×. The reads were aligned to the human reference genome (UCSC GRCh37/hg19) using the Burrows-Wheeler Aligner (BWA v0.7.15) BWA-MEM algorithm. Reads with low mapping quality scores were removed. Presumed PCR duplicates were identified using Picard's MarkDuplicates and removed. Local alignment optimization and base quality recalibration were performed using the Genome Analysis Toolkit (GATK). Single-nucleotide variants (SNVs) and InDels were identified with Mutect2, respectively, and saved in a variant call format (8). Variants were functionally annotated and filtered with ANNOVAR (http://annovar.openbioinformatics.org/en/latest), which provided built-in public databases (OMIM, InterVar, ClinVar, HGMD, Cosmic70, dbSNP, 1000G, ESP, ExAC, and gnomAD), and the HGMD Professional database (9). We identified a *de novo* variant (c.116 G>A, p.S39N) in exon 2 of *DNM1L*, which has not been reported previously. Furthermore, gene variation was validated using Sanger sequencing (Figure 1C). The conservation of the identified amino acid across different species and the protein 3D modeling for the *DNM1L*-wild and identified missense variant were shown in Figures 1D–F. The pathogenicity score was checked using the following online tools (Table 1).

**Table 1:** The pathogenicity prediction using different online tools.

**Predicting tools**	**Polyphen2**	**VariantTaster**	**SIFT**	**ACMG**
pathogenicity	probably	Disease	Deleterious	Pathogenic
	damage	causing		PS2+PM1+PM2+PP1+PP2+PP3

#### Treatment and Follow-Up

Benzhexol (0.5 mg BID) and Medopar (31.25 mg QID) were used to reduce muscle tension, and a cocktail therapy (VitB1 10 mg TID, VitC 0.5 g BID, VitE 100 mg QD, CoQ 10 mg TID and L-carnitine 100 mg BID) was administered to treat the primary illness, but none provided significant clinical benefit. At the last follow-up, the patient was 3 years and 4 months of age. He was 85 cm tall and weighed 8 kg, with a head circumference of 46 cm. He was admitted to our hospital with symptoms including spastic tetraparesis, no postural control, paroxysmal hypermyotonia and tremor, and severe cognitive impairment but no seizures. He could only follow a voice and light, occasionally respond to communication, and make vowel sounds, but he could not cry or laugh out loud.

### Literature Review

We searched for reported cases in the PubMed database using the terms “*DNM1L*” and “mitochondrial.” Thirty-five cases from 20 relevant references (5–7, 10–26) were retrieved; these included the first case reported by Waterham in 2007 and other cases reported from January 2015 to date. A total of 36 cases were analyzed together with the case reported by us. Fifteen males, 16 females, and two patients without reference to sex and three pedigrees (both male and female) were included. The median age of onset was 6 months (range, neonatal period: 9 years).

### Clinical Data Summary

Clinical manifestations included psychomotor retardation in 77.8% (28/36) of patients, limb paralysis in 66.7% (18/27), dystonia in 82.8% (24/29), and epilepsy in 59.4% (19/32). Other symptoms included ataxia in six cases, nystagmus in three cases, optic atrophy in six cases, dysarthria in four cases, microcephaly in four cases, pain insensitivity in two cases, and sensory and motor axonal neuropathy in two cases.

On auxiliary examination, 83.3% (25/30) of the cases had abnormalities in brain MR images, including abnormal hyperintensity in the cortex (10 cases), basal ganglia/thalamus (10 cases), subcortical white matter (three cases), brain atrophy (12 cases), lactate peak (four cases), and thinning/absence of the corpus callosum (three cases). Seventy-five percent (15/20) of the cases showed abnormalities in EEG, such as epileptic discharge or a weak background. Hyperlactacidemia was identified in 60.7% (17/28) of the cases, and decreased respiratory chain enzyme activity was detected in 28.6% (4/14) of cases. Muscle biopsy was performed in 22 cases, ragged red fibers were observed in four cases, and long tangled or swollen mitochondria were observed in 12 cases.

#### Prognosis

At the last follow-up, the 23 surviving patients had a median age of 7 years (2–27 years), whilst 12 had died with a median age at death of 1.5 years (from the neonatal period to 13 years). The median course of follow-up was 3.5 years (range, 7–24 years), and the median course of disease from illness to death was 1 year (range, 8–11.5 years). At the last follow-up, barring three families with optic atrophy as an isolated symptom, the rest had varying degrees of dyskinesia (four patients could walk with assistance, two could stand with assistance, one could sit alone, 11 could not control body position, and the rest were not mentioned), cognitive impairment, dystonia, or epileptic seizure.

#### Genetic Analysis

*De novo* variants were identified in 72.2% (26/36) of the cases, 13.9% (5/36) had heterozygous dominant variants, 11.1% (4/36) had compound heterozygous variants, and 2.8% (1/36) had homozygous variants. A total of 24 *DNM1L* variants were identified, including 21 missense variants, two deletion variants, and one duplication variant (Table 2).

**Table 2:** Related patient's information and variants in *DNM1L*.

**Patients**	**Country/District**	**Gender**	**Variant**		**Variant type**	**Reference**
Pedigrees 1–2	France	Pedigree 1:four male, four femalePedigree 2:six male, three female	c.5A>C	p.Glu2Ala	Heterozygous variant	Gerber et al. (18)
Patient 3	USA/Canada	Female	c.95G>C	p.G32A	*De novo*	Whitley et al. (22)
Patient 4–5(A pair of brothers)	Italy	two male	c.106A>G; c.346_347delGA	p.Ser36Gly;p.Glu116Lysfs*6	Compound heterozygous variants	Nasca et al. (5)
Patient 6	China	Male	c.116G>A	p.S39N	*De novo*	—
Patient 7–8(A pair of siblings)	Canada	FemaleMale	c.261dup; c.385_386del	p.Trp88Metfs*;p.Glu129Lys*6	Compound heterozygous variants	Yoon et al. (15)
Patient 9	Canada	Female	c.305C>T	p.T115M/T115M	Homozygous variants	Hogarth et al. (19)
Patient 10	Italy	Female	c.436G>A	p.D146N	*De novo*	Longo et al. (26)
Pedigree 11	France	three male, one female	c.575C>A	p.Ala192Glu	Heterozygous variant	Gerber et al. (18)
Patient 12	Italy	Female	c.668G>T	p.Gly223Val	*De novo*	Verrigni et al. (6)
Patient 13	USA	Male	c.1048G> A	p.G350R	Heterozygous variant (Maternal mosaic)	Chao et al. (11)
Patient 14–15	Italy Arab	FemaleMale	c.1084G>A	p.Gly362Ser	*De novo*	Verrigni et al. (6)Sheffer et al. (13)
Patient 16	Canada	Female	c.1085G>A	p.Gly362Asp	*De novo*	Vanstone et al. (14)
Patient 17	Italy	Male	c.1085G>A c.1535T>C	p.Gly362Aspp.lle512Thr	Heterozygous variant; the two variants were on the same maternal allele, c.1085G>A was present in the mother's blood DNA at a low level	Verrigni et al. (6)
Patient 18	Italy	Female	c.1109T>G	p.Phe370Cys	*De novo*	Verrigni et al. (6)
Patient 19	USA	Female	c.1135G> A	p.E379K	*De novo*	Chao et al. (11)
Patient 20–21	UK USA/Canada	FemaleFemale	c.1184C>A	p.A395D	*De novo*	Waterham et al. (10)Whitley et al. (22)
Patient 22–31	USA Italy USA /UK USA Turkey USA USA/Canada	two maleone maletwo not mentionedone maleone maleone femaleone male, one female	c.1207C>T	p.Arg403Cys	*De novo*	Fahrner et al. (12)Fahrner et al. (6)Ladds et al. (20)Ryan et al. (21)Schmid et al. (24)Nolan et al. (23)Whitley et al. (22)
Patient 32	Japan	Male	c.1217T>C	p.Leu406Ser	*De novo*	Zaha et al. (16)
Patient 33	USA	Female	c.1228G> A	p.E410K	*De novo*	Vandeleur et al. (25)
Patient 34	USA/Canada	Female	c.1292G>A	p.C431Y	*De novo*	Whitley et al. (22)
Patient 35	Spain	Female	c.1337G>T	p.Cys446Phe	*De novo*	Zaha et al. (16)
Patient 36	USA	Female	c.2072A> G	p.Tyr691Cys	*De novo*	Assia Batzir et al. (7)

### Correlation Between Domains and Phenotypes

#### Clinical Phenotype

The clinical manifestations of the GTPase domain group were milder than those of the middle domain group (Figure 1G, Table 3). The prevalence of psychomotor retardation and epilepsy in the GTPase domain group was lower than that in the middle-domain group (Psychomotor retardation: GTPase domain group, 50.0% [6/12] vs. middle domain group, 91.3% [21/23]; epilepsy: GTPase domain group, 8.3% [1/12] vs. middle domain group, 89.5% [17/19]); these differences were statistically significant (*P* < 0.05). In the five patients with heterozygous variants, three pedigrees with variants in the GTPase domain presented with optic atrophy as the isolated symptom, which was the mildest phenotype, and the family pedigrees were shown together with the segregation of the *DNM1L* variants in the affected individuals. The variants of the two other patients were located in the middle domain; one patient had a c.1048G>A, p.G350R variant, and variant analysis by Sanger sequencing suggested a low level (6–8%) of maternal mosaicism. The patient presented with developmental delay, hypotonia, status epilepticus, and elevated serum lactate levels, and he died of status epilepticus at the age of 5. Another patient had two heterozygous *DNM1L* variants (c.1085G>A, p.Gly362Asp; c.1535T>C, p.lle512Thr) on the same allele (maternal allele). His mother with the variant (c.1535T>C, p.lle512Thr) was healthy, but the other variant (c.1085G>A, p.Gly362Asp) was not present in either parent. However, further analysis of the *DNM1L* gene using a next-generation sequencing approach revealed that the variant (c.1085G>A, p.Gly362Asp) was present in the mother's blood DNA at a very low level. Furthermore, the patient's half-brother had died at the age of 3 years. Therefore, these findings supported maternal germline mosaicism for a dominant variant (c.1085G>A, p.Gly362Asp). The patient manifested developmental delay prior to the onset of illness. At 5 years and 5 months of age, he presented with partial motor status epilepticus after a viral illness. 4 months later, he presented with spastic quadriplegia and hyperkinesis but poor voluntary movements, no postural control, and severe cognitive impairment. However, there was no statistically significant difference in the survival rate in the different domain groups (log-rank test, *P* = 0.39, Figure 1H).

**Table 3:** The clinical characteristics in patients with *DNM1L* variants in different domains.

**Clinical data**		**GTPase domain**	**Middle domain**	***P*-value**
**%(n/n)or n**		**(*n* = 12)**	**(*n* = 23)**	
Age of onset		0.5 (0.1–5)	0.5 (0.1–9)	/
Sex	Male	44.4% (4/9)	52.4% (11/21)	1.000
	Female	55.6% (5/9)	47.6% (10/21)	
The last	Survive	83.3% (10/12)	56.5% (13/23)	0.149
follow-up	death	16.7% (2/12)	43.5% (10/23)	
Psychomotor retardation		50.0% (6/12)	91.3% (21/23)	0.011
Limb paralysis		66.7% (8/12)	64.3% (9/14)	1.000
Dependent ambulation		2	2	
Stand		–	1	
Sitting		–	1	
No postural control		6	5	
Dystonia		72.7% (8/11)	88.2% (15/17)	0.353
Epilepsy		8.3% (1/12)	89.5% (17/19)	0.000
Status epilepticus		1	12	
Ataxia		5	1	/
Nystagmus		1	1	/
Optic atrophy		4	1	/
Dysarthria		3	1	/
Microcephalus		1	3	/
Pain insensitivity		–	2	/
Sensory and motor axonal neuropathy		1	1	/
Abnormality in MR image		62.5% (5/8)	90.9% (20/22)	0.102
Hyperintense in cortex		1	9	/
Hyperintense in white matter		1	2	/
Hyperintense in basal ganlia/ hypothalamus		4	6	/
Cerebral atrophy		1	11	/
thinning/absence of corpus callosum		0	3	/
lactate peak		1	3	/
Abnormal EEG		20.0% (1/5)	92.9% (13/14)	0.006
Epileptiform discharge		1	12	
Weak background		–	7	
Hyperlactacidemia		57.1% (4/7)	65.0% (13/20)	0.661
decreased respiratory chain enzyme activity		0.0% (0/3)	36.4% (4/11)	0.505
Ragged red fibers		1	3	/
Abnormal morphology of mitochondria		5	6	/

*Data are shown as prevalence %(n/n) or n(number). “–” indicates that it was not mentioned in the literature. Statistical analysis included the chi-square test or Fisher's exact probability, as appropriate. “/” indicates that no statistical analysis was performed*.

Auxiliary examination revealed that 83.3% (25/30) of the cases had abnormalities in brain MR images; 62.5% (5/8) of the GTPase domain group vs. 90.9% (20/22) of the middle domain group (*P* = 0.102), with no statistically significant difference. Seventy-five percent (15/20) of the cases showed abnormality in EEG; 20.0% (1/5) of the GTPase domain group vs. 92.9% (13/14) of the middle domain group (*P* = 0.006), with statistically significant differences.

## Discussion

Mitochondrial diseases are a group of disorders with heterogeneous clinical and genetic characteristics caused by mitochondrial structural or function disturbances due to nuclear or mitochondrial DNA variations. There have been several studies on mitochondrial respiratory chain complex defects and mitochondrial diseases, but there are few reports on mitochondrial diseases caused by abnormal mitochondrial dynamics. Mitochondria are highly dynamic organelles that undergo continuous fission, fusion, and migration. The genes that encode these mitochondrial dynamic-related proteins are mitochondrial nuclear genes, and variants in these genes can lead to mitochondrial diseases: (1) mitochondrial membrane fusion abnormality: gene variants in *MFN2* encoding a GTPase joining adjacent mitochondria leads to Charcot–Marie–Tooth disease type 2A and hereditary motor and sensory neuropathy VI, and optic atrophy or Leigh-like infantile encephalopathies are caused by variants in *OPA1*, which encodes a GTPase that mediates the fusion of the inner mitochondrial membrane (5, 6). (2) Mitochondrial fission abnormality: gene variants in *DNM1L* involving mitochondrial fission lead to encephalopathy; gene variants in the mitochondrial fission factor (*MFF*), the receptor gene of *DNM1L*, cause Leigh-like encephalopathy (27). (3) Mitochondrial migrating abnormality: gene variants in *Miro* are associated with Parkinson's disease, Alzheimer's disease, and amyotrophic lateral sclerosis (28, 29).

*DNM1L*, encoding DRP1, is located in chromosomal region 12p11.21. DRP1 is a highly conserved GTPase that mediates mitochondrial fission and contains an *N*-terminal GTPase domain, a middle domain, a non-conserved variable domain, and a C-terminal GTPase effector domain (GED) (19). DRP-1, a cytosolic protein that can be recruited to the mitochondria in response to cellular signals, is the most important mediator of mitochondrial and peroxisomal division. Different sites in different domains can bind to the receptor on the mitochondrial outer membrane. The GTPase domain is responsible for binding to receptors on the outer membrane of the mitochondria. The middle domain mainly mediates the oligomerization of DRP1, with the fission process executed by DRP1 forming a concentric ring-like structure around the scission site, followed by GTPase-dependent constriction (15, 19). GED stimulates GTPase activity and mediates the formation and stability of the DRP1 homodimer complex (7). *DNM1L* variants impair the oligomerization of DRP1, thereby affecting the contraction of GTPase and leading disrupting mitochondrial fission.

*DNM1L*-related mitochondrial diseases are rarely reported, and limited gene variants of *DNM1L* are included in the Human Gene Mutation Database. We identified a *DNM1L* variant that had not previously been reported, and enriched the genetic information from the Chinese population to improve the understanding of rare diseases. The clinical phenotype of this patient was consistent with that previously reported.

The clinical symptoms of *DNM1L-*related mitochondrial disease vary. The median age of onset was 6 months (from the neonatal period to 9 years), and the main symptoms included psychomotor retardation, limb paralysis, dystonia (hypermyotonia or hypomyotonia), epilepsy, and other symptoms, including ataxia, nystagmus, optic atrophy, dysarthria, microcephaly, pain insensitivity, and sensory and motor axonal neuropathy.

Most *DNM1L* variants were located in the middle domain (*n* = 23) and GTPase domain (*n* = 12) of DRP1. The severity of clinical symptoms was related to the domains in which the *DNM1L* variants were located. The clinical symptoms of the GTPase domain group were milder than those in the middle domain group. In the GTPase domain group, three families presented with optic atrophy as an isolated symptom, which was the mildest phenotype, and the family pedigrees were shown with the segregation of *DNM1L* variants in the affected individuals. The prevalence of psychomotor retardation and epilepsy in the GTPase domain group was lower than that in the middle domain group, and the proportion of status epilepticus and refractory epilepsy was higher in the middle domain group. However, there was no significant difference in occurrence of limb paralysis or dystonia between the two groups. The variant c.116G>A (p.S39N) in our patient was located in the GTPase domain of DRP1, and he presented with psychomotor retardation, limb paralysis, paroxysmal hypermyotonia, and limb tremors, but no epileptic seizures were observed during the 3-year-follow-up. This highlights the importance of the location of variants in determining clinical phenotypes.

As DRP1 is a cytoplasmic protein, it is recruited to the outer mitochondrial membrane by specific receptors. These include mitochondrial fission 1, MFF, and mitochondrial dynamics proteins of 49 and 51 kDa (MID49, MID51), which can all independently recruit DRP1 to mediate mitochondrial division (12, 30, 31). After binding with its receptors, DRP1 polymerizes to a concentric ring-like structure, encircles the mitochondria, and causes mechanochemical contraction of mitochondria. Taking MID49 as example, each chain of DRP1 binds MID49 through four different surfaces in different domains, and each MID49 in turn binds four DRP1, generating a huge interaction network (31). Variants in the GTPase domain can destroy the binding of the GTPase domain, while there are multiple interaction sites in oligomers that may hinder their cohesion; thus, the clinical phenotype is relatively milder (19) (Kalia et al., 2018). In addition to *de novo* variants, reported homozygous variants and compound heterozygous variants were all located in the GTPase domain, while recessive genes included null alleles and hypomorphic alleles, which could explain the different clinical phenotypes (5, 21). However, variations in the middle domain can impair the oligomerization of DRP1 and cause mitochondrial fission defects; therefore, the clinical phenotype is typically worse.

The prognosis of *DNM1L*-related mitochondrial diseases is poor. At the last follow-up, only 23 patients had survived, with 12 deaths with at a median age of 1.5 years. Although there was no statistically significant difference in the survival rate in different domains, patients with variants in the GTPase domain had a relatively better prognosis and could survive longer, and three families with variants in the GTPase domain showed isolated manifestations of optic atrophy. The remaining patients had varying degrees of dyskinesia, cognitive impairment, dystonia, or epileptic seizures.

By reviewing the relevant literature, the most common symptoms of *DNM1L*-related mitochondrial diseases were summarized; based on these finding, *DNM1L*-related mitochondrial diseases should be considered in the early stages of severe encephalopathy, including neonatal fatal diseases, psychomotor retardation, motor disorders, cognitive disorders, and epilepsy. The variant sites of *DNM1L* were summarized, and the understanding of the *DNM1L* gene profile was expanded.

## Ethics Statement

The studies involving human participants were reviewed and approved by Medical Ethics Committee of Tianjin Children's Hospital, Tianjin Children's Hospital. Written informed consent to participate in this study was provided by the participants' legal guardian/next of kin. Written informed consent was obtained from the individual(s), and minor(s)' legal guardian/next of kin, for the publication of any potentially identifiable images or data included in this article.

## Author Contributions

ZZ and DL retrieved and summarized the related literature. XinL wrote the review article. ML created the figures. QL provided statistical analysis. XiaL provided the medical records. PZ revised the review article. All authors contributed to the article and approved the submitted version.

## Conflict of Interest

The authors declare that the research was conducted in the absence of any commercial or financial relationships that could be construed as a potential conflict of interest.
